# 574. High Acceptance and Rapid Implementation of COVID-19 Vaccine in a Public HIV Clinic in Northern California: An Initial Analysis of Social Determinants

**DOI:** 10.1093/ofid/ofab466.772

**Published:** 2021-12-04

**Authors:** Jennifer Lin, Frank Oi-Shan Wong, Christopher Thibodeaux, Moon Choi-McInturff, Aracely Tamayo, Vivian Levy

**Affiliations:** San Mateo County Health System, San Mateo, CA

## Abstract

**Background:**

Safety net HIV providers face operational challenges during the COVID pandemic with services often transformed to telehealth. HIV infected persons are a priority population for SARS CoV-2 vaccination. Medical mistrust of COVID vaccines has been cited as a contributor to vaccine hesitancy. Data on efficient and successful vaccination efforts of HIV infected persons in safety net health systems is needed. In San Mateo County, Latino persons comprised 42% of all COVID cases, Whites 16%, and African Americans 2%.

**Methods:**

SARS CoV2 vaccination with BNT162b2 (Pfizer–BioNTech), mRNA-1273 (Moderna) or Ad26.COV2.S (Janssen) vaccine were offered beginning February 2, 2021 through May 28, 2021 in a northern California public County HIV clinic. Clinic patients were contacted by bilingual English/Spanish speaking HIV clinic staff and appointments scheduled at County affiliated vaccination sites. Clinic staff followed up by phone with patients who did not initially accept vaccine. We calculate the percentage of patients who completed vaccine series and use multivariable logistic regression analysis to estimate the odds of series completion by patient race/ethnicity, gender and age.

**Results:**

Virtually all, 95% (349/365) of HIV patients in our County HIV clinic were offered vaccine during a 17 week period. Among those, 86% (313/365) accepted and received at least one dose and 80% completed the series (292/365) at time of this analysis. Janssen vaccine was given to only 2% (7/313) patients. Series completion was highest among Latinos and Asians. Latinos had the highest odds of vaccine series completion (OR = 4.12; 95% CI 1.71 - 9.93).

COVID-19 Vaccine Series Completion in a California Public HIV Clinic by Race/Ethnicity, Age and Sexual Orientation, n=364

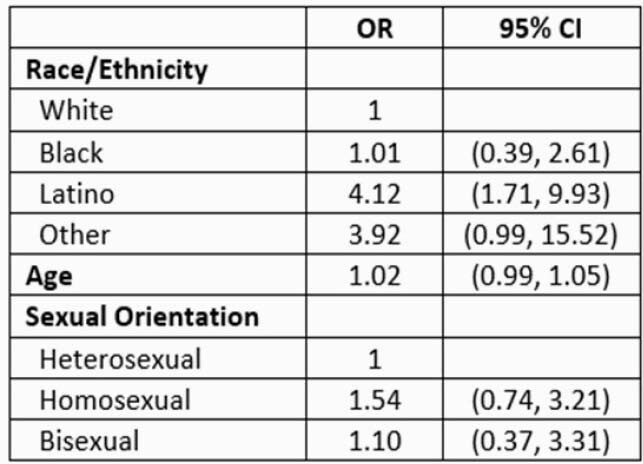

**Conclusion:**

HIV patients offered SARS CoV2 vaccine by County HIV clinic staff with established patient care relationships had high vaccine acceptance (80%), comparable to 68% series completion in the county overall and 56% in the health equity quartile county census tracts. Latino HIV infected persons were most likely to complete the COVID vaccine series. Ryan White funded HIV clinics are ideal hubs to coordinate HIV patient COVID vaccination efforts. Adding COVID vaccine completion to HIV clinic performance measures would likely be beneficial.

**Disclosures:**

**All Authors**: No reported disclosures

